# Chemokines as Cancer Vaccine Adjuvants 

**DOI:** 10.3390/vaccines1040444

**Published:** 2013-10-16

**Authors:** Iuliana D. Bobanga, Agne Petrosiute, Alex Y. Huang

**Affiliations:** 1 Departments of General Surgery, School of Medicine, University Hospital Case Medical Center/Case Western Reserve University, Cleveland, OH 44106, USA; 2 Departments of Pediatrics, School of Medicine, University Hospital Case Medical Center/Case Western Reserve University, Cleveland, OH 44106, USA

**Keywords:** chemokines, dendritic cell, immunotherapy, cancer vaccine, adjuvant

## Abstract

We are witnessing a new era of immune-mediated cancer therapies and vaccine development. As the field of cancer vaccines advances into clinical trials, overcoming low immunogenicity is a limiting step in achieving full success of this therapeutic approach. Recent discoveries in the many biological roles of chemokines in tumor immunology allow their exploitation in enhancing recruitment of antigen presenting cells (APCs) and effector cells to appropriate anatomical sites. This knowledge, combined with advances in gene therapy and virology, allows researchers to employ chemokines as potential vaccine adjuvants. This review will focus on recent murine and human studies that use chemokines as therapeutic anti-cancer vaccine adjuvants.

## 1. Introduction

Chemokines are a group of related chemoattractant peptides that are essential regulators of the immune system, both during homeostatic and inflammatory conditions. Over the last few decades, chemokines are found to be involved in almost every aspect of tumorigenesis and antitumor immunity [1]. While a function of chemokines is to regulate lymphocyte trafficking, the view that chemokines act simply as “chemotactic cytokines” has evolved to include the many critical roles they play in regulating innate and adaptive immune responses. For example, in addition to chemotaxis, chemokines modulate lymphocyte development, priming and effector function [2] and play a critical role in immune surveillance. Some inflammatory chemokines have proven essential in memory T cell generation [3]. In the context of cancer, the chemokine-chemokine receptor system plays paradoxical roles. On one hand, the chemokine network is used by tumors to evade immune surveillance, resist apoptosis, and metastasize. On the other hand, the chemokine system also plays a crucial role in the induction of antitumor immune responses and optimal effector function regulation of immune cells [1,4,5].

To date, there are more than 50 chemokines and 18 chemokine receptors identified [6]. These molecules are classified into four families (CC, CXC, C, and CX3C) based on the way the first two conserved cysteine residues are arranged, creating a structural motif [6]. Two nomenclature systems are often interchangeably sited in the literature: the name at the time of discovery, and the systematic nomenclature as described in Table 1 [6]. For consistency, this review will henceforth use the systematic nomenclature. Most chemokines bind to more than one receptor, while most receptors also display overlapping ligand specificity [5]. Functionally, chemokines are described as inflammatory (inducible) or homeostasis (constitutive) based on their pathophysiological activities. Inflammatory chemokines are secreted in inflamed tissues by resident and infiltrated cells after stimulation by pro-inflammatory cytokines or during contact with pathogens. They specialize in the recruitment of effector cells, particularly monocytes, granulocytes, and effector T cells to sites of inflammation, tissue destruction, or tumor microenvironment (TME). Homeostatic chemokines are constitutively produced and regulate physiologic trafficking of immune cells during hematopoiesis, antigen sampling in secondary lymphoid tissue and immune surveillance. Some chemokines are also defined as angiogenic or angiostatic based on their role in promoting or suppressing tissue neovascularization, respectively [7].

**Table 1:** Chemokine nomenclature, corresponding receptors, and category based on function. Adopted from [6,7]. Chemokines used as adjuvants for vaccines in murine and human studies are highlighted in color.

Chemokine standard name	Chemokine discovery name	Corresponding receptor	Functional category
CXCL1	GROα/MGSA-α	CXCR2, CXCR1	inflammatory and angiogenic
CXCL2	GROβ/MGSA-β	CXCR2	inflammatory and angiogenic
CXCL3	GROγ/MGSA-γ	CXCR2	inflammatory and angiogenic
CXCL4	PF4	CXCR3-B	angiostatic
CXCL5	ENA-78	CXCR2	inflammatory and angiogenic
CXCL6	GCP-2	CXCR1, CXCR2	inflammatory and angiogenic
CXCL7	NAP-2	CXCR1, CXCR2	inflammatory and angiogenic
CXCL8	IL-8	CXCR1, CXCR2	inflammatory and angiogenic
CXCL9	MIG	CXCR3	inflammatory and angiostatic
CXCL10	IP-10	CXCR3	inflammatory and angiostatic
CXCL11	I-TAC	CXCR3, CXCR7	inflammatory and angiostatic
CXCL12	SDF-1	CXCR4, CXCR7	homeostatic
CXCL13	BCA-1	CXCR5, CXCR3	homeostatic
CXCL14	BRAK/bolekine	unknown	Homeostatic
CXCL16	SR-PSOX	CXCR6	inflammatory
CXCL17	DMC	unknown	homeostatic
XCL1	lymphotactin/SCM-1α/ATAC	XCR1	inflammatory and homeostatic
XCL2	SCM-1β	XCR1	inflammatory and homeostatic
CX3CL1	Fractalkine	CX3CR1	inflammatory, homeostatic and angiogenic
CCL1	I-309	CCR8	inflammatory and angiogenic
CCL2	MCP-1/MCAF/TDCF	CCR2	inflammatory and angiogenic
CCL3	MIP-1α/LD78α	CCR1, CCR5	inflammatory
CCL3L1	LD78β	CCR1, CCR5	inflammatory
CCL4	MIP-1β	CCR5	inflammatory
CCL5	RANTES	CCR1, CCR3, CCR5	inflammatory
CCL7	MCP-3	CCR1, CCR2, CCR3	inflammatory
CCL8	MCP-2	CCR3, CCR5	inflammatory
CCL11	Eotaxin-1	CCR3	inflammatory, homeostatic and angiogenic
CCL13	MCP-4	CCR2, CCR3	inflammatory
CCL14	HCC-1	CCR1, CCR3, CCR5	
CCL15	HCC-2/Lkn-1/MIP-1δ	CCR1, CCR3	
CCL16	HCC-4/LEC/LCC-1	CCR1, CCR2, CCR5	
CCL17	TARC	CCR4	inflammatory and homeostatic
CCL18	DC-CK1/PACRC/AMAC-1	unknown	homeostatic
CCL19	MIP-3β/ELC/exodus-3	CCR7	homeostatic
CCL20	MIP-3α/LARC/exodus-1	CCR6	inflammatory and homeostatic
CCL21	6Ckine/SLC/exodus-2	CCR7	homeostatic
CCL22	MDC/STCP-1	CCR4	inflammatory and homeostatic
CCL23	MPIF-1/CKβ8/CKβ8-1	CCR1	
CCL24	Eotaxin-2/MPIF-2	CCR3	homeostatic
CCL25	TECK	CCR9	homeostatic
CCL26	Eotaxin-3	CCR3	inflammatory
CCL27	CTACK/ILC	CCR10	homeostatic
CCL28	MEC	CCR3, CCR10	homeostatic

## 2. Chemokines Modify Effector Cell and APC Function

Development of an effective antitumor immune response depends upon the unified interaction of immunocompetent cells and their trafficking pattern between the tumor site and secondary lymphoid organs (e.g., lymph nodes (LNs) and spleen). This trafficking pattern is coordinated by chemokines acting through their corresponding receptors [5]. Dendritic cells (DCs) are professional APCs responsible for initiation or inhibition of immune responses by priming or tolerizing T cells [8]. Chemokines play a key role in the migration and recruitment of DCs. DC precursors in the peripheral blood migrate into peripheral tissues and differentiate to become immature DCs (iDCs), characterized by high phagocytic ability and increased levels of MHC molecules, but a lack of costimulatory molecules [9]. iDCs are guided by inflammatory chemokines (CCL2, CCL3, CCL4, CCL5, CCL7, and CCL20) to migrate to sites of inflammation or tissue damage, where they pick up antigen, upregulate costimulatory molecules, and become activated, mature DCs (mDCs). This chemotactic migration of iDCs within tissue is related to their expression of CCR1, CCR2, CCR5, and CCR6, while mDCs downregulate these chemokine receptors and upregulate CCR7 [1,9,10]. It is the constitutive expression of the CCR7 ligands CCL19 and CCL21 by the stromal cells in the T cell zones that guides the mature and antigen-loaded mDCs to secondary lymphoid organs, where they present processed antigens to the CCR7-expressing naïve or central memory T cells [1,9,10,11].

To become effective tumor-associated antigen (TAA)-specific killer cells, cytotoxic T lymphocytes (CTLs) require effective priming by DCs, which in turn require licensing by CD4^+^ T cells [12]. For this purpose, naïve CD8^+^ and CD4^+^ T cells, expressing CCR7, continuously scan the surface of DCs in secondary lymphoid organs in search for their rare cognate antigen [13]. Several chemokines are found to be critical to this process. CCL3 and CCL4 secreted by DCs in inflamed lymph nodes help guide naive CD8^+^ T cells expressing CCR5 to sites where CD4^+^ and CD8^+^ T cells are actively interacting with antigen-presenting DCs. The ternary cluster formed by the naïve CD8^+^ T cell, the CD4^+^ T cell and the DC enhances memory CD8^+^ T cell generation [3,14]. Additionally, mDCs secrete CCL19 to increase scanning behavior and antigenic response by naïve CD4^+^ T cell [15]. Upon TCR-MHC engagement, chemokine receptors also act as co-stimulatory molecules in the immunological synapse to further enhance signal transduction between the T cells and the APCs [16]. Following priming and T cell expansion, a change in the pattern of chemokine receptor expression is required for the redistribution of T cells from the secondary lymphoid organ back towards the target tissue. Once effector T cells have differentiated, they downregulate CCR7 and upregulate receptors specific to chemokines expressed in target tissues, such as CCR1, CCR2, CCR3, CCR5, and CXCR3 [5,17]. Thus, chemokines are critical in regulating the traffic of immune cells between the TME and draining LNs, as well as enhancing differentiation of naïve T cells into TAA-specific CTLs.

Effective cancer vaccines are designed to boost host adaptive immunity from a functionally tolerized state against cancer cells to one that can mount a functionally competent, tumor-specific, CD4^+^ and CD8^+^ effector and memory T cell-mediated immune response. As such, adjuvants such as Toll-like receptor (TLR) agonists (for example, CpG and PolyI:C) have been used in cancer vaccine to achieve this effect [18,19,20]. Due to their multifaceted roles in tumor immunology, chemokines represent another class of molecules that are attractive candidates for manipulation in cancer immunotherapy. Various chemokine-based tumor immunotherapies have been investigated, most of them in early preclinical models. A challenge to investigators in this research arena is that chemokines have been shown to be pro-tumorigenic in some tumor systems while anti-tumorigenic in others [1,4,8]. Some strategies target the pro-tumorigenic roles of chemokines by inhibiting chemokines and chemokine receptors that promote angiogenesis, tumor growth, and metastasis in certain tumor models [8]. Other strategies that deliver chemokines within the tumor microenvironment (TME) have been associated with enhanced antitumor immune response, increased angiostatic effect, low recurrence rate and increased patient survival [5]. In this light, immune-based cancer vaccines are strategies that can benefit from the addition of chemokines. These strategies vary based on the mode of tumor antigen loading unto professional APCs (e.g., peptide/protein-pulsed DC vaccines and peptide/DNA vaccines) [21]. This review will focus on the current use of chemokines as cancer vaccine enhancers. 

## 3. Chemokines as Adjuvants for Cancer Vaccines

The main goal of cancer vaccines is to elicit a tumor-specific adaptive immune response by activating CD8^+^ cytotoxic T lymphocytes for tumor cell lysis and Th1 CD4^+^ T cells to enhance CTL activity [1,22,23]. Cancer vaccines are likely to be most effective in a setting of minimal residual disease (MRD), once the bulky tumor has been reduced by other therapeutic modalities [1]. Since the FDA has approved the first therapeutic cancer vaccine for metastatic castrate-resistant prostate cancer, a wide range of cancer vaccines are now undergoing evaluation in Phase II and III clinical trials [23]. Various cancer vaccines are currently under investigation in clinical trials, including peptide, viral vector, whole cell/lysate, genetically modified tumor cell, and DC-based vaccines [21,23]. Each of these vaccine groups has their unique properties that create specific advantages and challenges. The common disadvantage in all cancer vaccines is the realization that TAA presentation alone is not sufficient to create the most efficient tumor eradication and memory response. Therefore researchers now focus on various techniques to enhance TAA immunogenicity and vaccine efficacy. As described below, chemokines can be useful adjuvants in different vaccine settings. The choice of chemokines varies from homeostatic (e.g., CCL19 and CCL21) to inflammatory (e.g., CCL3 and CCL5). The major contribution provided by chemokines is more robust recruitment of relevant immune cells towards tumor recognition, immune priming, and killing. These discoveries lead to several murine cancer vaccine studies with chemokines as additives (summarized in Table 2), and provided the scientific rationale for subsequent Phase I and Phase II clinical trials (summarized in Table 3). 

**Table 2:** Vaccine approaches incorporating various chemokines.

Vaccine Approach	Chemokine Approach	Cancer Type	Murine or Human	Reference
DC Vaccines	Use of CCL3 and CCL20 to improve DCs collection	Gastric Cancer	Murine	[24]
	XCL1 + gp100 DC vaccine	Melanoma	Murine	[25]
	Pre-treatment of DCs with CCL3	Melanoma	Murine	[26]
	Whole cell tumor lysate-pulsed DC vaccine transfected with CXCL10 pDNA	Glioma	Murine	[27]
	Insertion of CXCL10 gene into DCs	Cervical Cancer	Murine	[28]
	Whole cell tumor lysate-pulsed DC vaccine transfected with CCL21	Prostate Cancer	Murine	[29]
	Conditioning DC vaccine site with irradiated CCL20-expressing tumor cells		Murine	[30]
	DCs transfected with CCL21 gene	Hepatocellular Carcinoma	Murine	[31]
	DCs pulsed with whole tumor lysate and transfected with CXCL10 plasmid	Prostate	Murine	[32]
	Whole cell tumor lysate-pulsed DC vaccine combined with CCL5-containing vaccinia	Colon Cancer	Murine	[33]
	Intratumoral administration of gene-modified bone marrow DCs transduced with adenoviral vector expressing CCL21	Lung Cancer	Murine	[34]
	βgal pDNA * + CCL19 pDNA	Fibrosarcoma Lymphoma	Murine	[35]
	Her2/neu pDNA + CCL19 pDNA	Breast	Murine	[36]
	TERT DNA vacccine primed with CCL21	Breast	Murine	[37]
DNA Vaccines	Ova pDNA + CCL5-Ig pDNA	Lymphoma	Murine	[38]
	Her2/neu pDNA + CCL21 pDNA	Breast	Murine	[39]
	Ova pDNA + CX3CL1-Ig DNA	Lymphoma	Murine	[40]
	pCCL21&-HP (encodes for Her2/neu + p53)-Fc construct	Melanoma	Murine	[41]
	pCCL21-E7-Fc	Cervical Cancer	Murine	[42]
	pCCL21-3P-Fc	Melanoma	Murine	[43]
	CCL21 + TRP DNA vaccine	Melanoma	Murine	[44]
	CCL5pDNA + gp100 pDNA vaccine, with CCL5 + hgp100 viral vector boost	Melanoma	Murine	[45]
	CCL21 pDNA + hgp100 pDNA +/− IL2	Melanoma	Murine	[46]
Whole Cell/Lysate or Gene Modified Cancer Cells	CCL21-expressing tumor cells	Melanoma	Murine	[47]
	CCL3+ IL2 or CCL3+ GMCSF	Leukemia/lymphoma	Murine	[48]
	B16F0 transfected with pCCL21-3p-Fc	Melanoma	Murine	[49]
	GMCSF-producing WEHI3B with recombinant CCL17 or CCL5	Murine Myelomonocytic Leukemia	Murine	[50]
	Glioma cell vaccine expressing CCL3 and GM-CSF	Glioma	Murine	[51]
	IL2 + GMCSF expressing Meth A and HM-1 tumor cells co-transfected with CCL21, CCL19 and CXCL12	Fibrosarcoma and Ovarian Cancer	Murine	[52]
TAA-Chemokines	Fusion of CCL7, CCL20, CXCL10 to TAA	B Cell Lymphoma	Murine	[53,54]

* pDNA, plasmid DNA; ^&^ pCCL21, plasmid DNA encoding CCL21.

**Table 3:** Clinical trials using chemokines as cancer vaccine adjuvants.

Type of vaccine	Trial description	Phase	Cancer Type	Status	Published?
DC	Intradermal injection of adenovirus-CCL21 transduced class I peptide-pulsed DCs [55]	Phase I	Melanoma	closed	no
	Intratumoral autologous DC-adenovirus CCL21 vaccine [56,57]	Phase I	Stage IIIB-IV or recurrent Non-Small Cell Lung Cancer	open	no
Genetically-modified Cancer Cells	Combination immunotherapy of GM.CD40L * vaccine with CCL21 [58]	Phase I	Lung Cancer	open	no
	Gene-modified tumor cells for relapsed/refractory disease (CYCHE) [59]	Phase I	Neuroblastoma	completed	no
	A phase I/II study of immunization with XCL1 and IL-2 gene modified tumor vaccine (CHESAT) [60]	Phase I/II	Neuroblastoma	open	no
	Allogeneic tumor cells for relapsed/refractory disease (CYCHEALL) [61]	Phase I	Neuroblastoma	open	no
TDNA vaccines	Phase I study for asymptomatic Phase disease with DNA vaccines encoding antigen-chemokine fusion [62]	Phase I	Asymptomatic Phase Lympho-plasmacytic Lymphoma	Not yet open	no

* GM.CD40L, genes encoding GM-CSF and CD40L.

### 3.1. Use of Chemokines to Enhance DC Vaccines: A Field Moving towards Phase I-II Clinical Trials

DCs are potent APCs that are capable of activating naive T cells and generating strong anti-tumor immunity [63,64]. iDCs can efficiently internalize antigen and, subsequently, process and present antigen peptides in conjunction with major histocompatibility complex (MHC) class I and II molecules to T lymphocytes. However, concerns have been raised regarding the use of iDCs in clinical trials since they have been associated with inducing T cell tolerance [64]. However, mDCs have a higher expression of MHC and costimulatory molecules after activation by danger signals in the periphery, and are therefore better equipped to activate antigen-specific T cells in secondary lymphoid organs. For this reason, mDCs loaded with TAAs *in vitro* have found clinical applications. Phase II studies have been conducted to evaluate the effectiveness of DC-based vaccines using various strategies (protein-pulsed, peptide-pulsed, or viral-vector infected DCs) to treat patients with prostate cancer, colorectal cancer, melanoma, glioma, and other cancers [21,23]. Of these approaches, a major challenge is that these vaccines do not always result in robust T cell activation, tumor killing by effector T cells, or generation of memory T cells. A reason for this is insufficient physical contacts among relevant immune cell types for optimal immune response generation. For these reasons, chemokines have been added to DC vaccines in an effort to improve antigen presentation and immune cell recruitment. In addition, chemokines have also been used to enhance DC recruitment *in vivo* for subsequent *in vitro* expansion. For example, He *et al.* showed that intravenous injection of CCL3 and CCL20 prior to DC collection improved recruitment of DCs. Subsequent transduction of those DCs with the melanoma TAA MAGE-1 gene resulted in improved melanoma tumor rejection *ex vivo* and *in vivo* [24]. In another study, CCL3 pre-treatment of mice resulted in the recruitment of more effective DCs in the peripheral blood. These DCs expressed a higher level of CCR7, displayed a more significant chemotactic response towards secondary lymphoid tissue, and generated a stronger CTL responses resulting in enhanced rejection of melanoma [26]. 

An attractive approach to enhance DC vaccine efficacy is to combine DCs with plasmid DNA (pDNA) encoding specific chemokines. Jiang *et al.* undertook such an approach by administering DCs pulsed with glioma cell line (GL261) lysate subcutaneously (SQ) into mice bearing glioma tumor [27]. A cohort of mice also received a plasmid encoding CXCL10 (pcDNA3.1-mIP-10) at the same vaccination site. As CXCL10 is a chemokine that has both anti-angiogenic and T cell recruitment properties into the CNS [65], mice receiving combination therapy had significantly improved survival rates (60% *vs.* 0%). A different group of researchers has attempted retroviral introduction of the CXCL10 gene into DCs and observed improved CD8^+^ T cell response and tumor rejection [28]. Li *et al.* pulsed bone marrow-derived DCs with murine prostate tumor lysate and transfected these cells with a plasmid vector encoding for CXCL10 [32]. Tumor rejection and survival was improved compared to mice receiving pulsed DCs or non-pulsed DCs with CXCL10 gene alone. 

CCL21 has also been implemented in DC vaccine strategies. Although considered to be a homeostatic chemokine, CCL21 influences T cell migration to secondary lymphoid organs during inflammation and enhances the Th1 T cell response [66]. Liang *et al.* transfected murine iDCs with the CCL21 gene using the recombinant adeno-associated virus serotype 2 (rAAV2) as a gene delivery vector [31]. When CCL21-transfected DCs were injected intratumorally in mice bearing hepatocellular carcinoma (HCC), mice exhibited delayed tumor progression, increased intratumoral T cell infiltration and overall improved survival. Yang *et al*. took a similar approach by transducing DCs with adenoviral vector encoding the CCL21 gene. Their data again showed better tumor eradication and tumor-protective immunity in the mouse cohort receiving CCL21-expressing DCs intratumorally [34]. Another study not only introduced CCL21 gene-encoding plasmid (pAAV-IRES-hrGFP/SLC) into bone marrow-derived DCs but also pulsed DCs with whole tumor lysate and then injected the construct into tumor-bearing mice with similar efficacy [29].

CCL20 was recently shown to direct iDC migration and is postulated to play a role in tumor immunotherapy [67]. SQ injection of irradiated tumor cells expressing CCL20, followed by a second vaccination of DCs pulsed with irradiated tumor cells at the same injection site resulted in significantly more robust tumor rejection than DC vaccine alone [30].

XCL1 is a chemokine that has shown the ability to attract effector cells (NK cells and CD8^+^ cells) and has been tested as a DC vaccine enhancer [68]. Xia *et al.* immunized mice with DCs co-transfected with XCL1 and melanoma antigen gp100 (XCL1/gp100-DC) using an adenoviral vector. Their results showed enhanced effects of CTL and NK cell activation and increased production of IL-2 and interferon-gamma. The XCL1/gp100-DC immunized mice exhibited resistance to tumor challenge more effectively compared to controls [25]. 

CCL5 is one of the central chemokines that regulates T cell migration towards sites of tissue injury and inflammation, as well as Th1 differentiation [69]. CCL5 has been tested in murine models as adjuvant therapy for tumor lysate-pulsed DC vaccines. Mice received tumor lysate-pulsed DC vaccine followed two days later by intraperitoneal (IP) injection of CCL5-expressing recombinant vaccinia virus [33] showed a significant reduction in rates of tumor growth and increased survival, which correlated with increased immune cell infiltration into tumor sites.

CCL19 is a potent inducer of T cell proliferation [70]. To bypass the labor-intensive process of isolating, expanding and loading DCs from individual patients *ex vivo*, Kumamoto *et al*. developed an approach to entrap epidermal Langerhans Cells (LCs) *in situ* and load them with TAAs [71]. They used subcutaneously (SQ) implanted CCL19-coated polymer rods to create a LC-attracting chemokine gradient during their migration from the epidermis to the draining LN. The entrapped LCs were antigen-loaded *in situ* by co-implantation of a second polymer rod releasing tumor-associated antigens. Once loaded with TAA *in situ*, CCL19 administration allowed LCs emigration from the epidermis to the draining LN to activate a strong antigen-specific CTL response [71]. 

These preclinical investigations lead researchers to successfully transduce CCL21-expressing human DCs [72], setting the ground work for future clinical trial development. A recently closed Phase I clinical trial in melanoma applied intradermal injections of adenovirus-CCL21 transduced class I peptide-pulsed DCs [55]. Dose-escalation studies of intratumoral autologous DC-adenovirus CCL21 vaccine in patients with advanced lung cancer are also currently open [56,57] (Table 3).

### 3.2. Chemokine Adjuvants to DNA Vaccines

DNA vaccines encompass DNA constructs that encode TAAs. Once administered SQ or intramuscularly (IM), DNA constructs are taken up by local cells, including APCs, that then express the TAAs on the cell surface in conjunction with MHC class I molecules. This TAA presentation ultimately leads to T cells response against TAA and therefore the tumor cells [73]. The use of DNA vaccines in cancer immunotherapy has many advantages (e.g., less costly, vastly available, safe, lack of autoimmunity, and less potential for rejection) [74]. However, the main challenge of such vaccine approach is their low immunogenicity [75]. 

As discussed above, CCL19 is a potent inducer of T cell proliferation [70], a feature that prompted trials of its use as an adjuvant for DNA vaccination in murine models [35]. Westermann’s group compared how mice-bearing tumors responded to vaccine with plasmid DNA (pDNA) encoding tumor DNA alone or vaccine with tumor DNA and CCL pDNA. Co-expression of pDNA encoding CCL19 and tumor antigen resulted in enhanced Th1 immune response and increased CD8^+^ T cell infiltration in the tumor bed. Similar experiments were conducted by injecting tumor-bearing mice IM with pDNA encoding Her2/neu with or without CCL19 pDNA [36]. Again, mice injected with both Her2/neu pDNA and CCL19 pDNA had substantially improved tumor protection (58% *versus* 22% tumor-free incidence). Similar results were obtained with CCL21 pDNA [39]. As CCL21 is another potent inducer of T cell proliferation, Yamano *et al.* injected CCL21 into mice at various time points before and after vaccination with TRP vaccine and showed CCL21 enhanced responses best when it was administered into the vaccine bed 24 hours prior to TRP DNA injection [44]. Another study tested CCL21 administration 24 hours before cTERT DNA vaccine [37]. Again, results showed significantly improved anti-TERT cell immunity in mice that received CCL21 chemokine compared to vaccine alone. Incorporating plasmid DNA encoding CCL21 gene (pCCL21) into a DNA vaccine construct containing fused common Her-2/neu and p53 (HP) to the Fc portion of IgG improves MHC II class presentation [41]. Injection of the end-product construct pCCL21-HP-Fc into melanoma-bearing mice resulted in improved tumor free survival (40% *vs.* 0% at 45days when compared to Fc controls) and better protection against subsequent tumor re-challenge. Similar DNA vaccine constructs encoding a single tumor antigen-E7 (pCCL21-E7-Fc), or multiple epitopes (pCCL21-3P-Fc), also showed improved tumor rejection and memory T cell generation in both cases [42,43]. 

The chemokine CX3CL1 contains chemoattractant properties for CTLs, NK cells, and macrophages [76], and was evaluated in pre-clinical models as a DNA vaccine adjuvant. DNA vaccine co-expressing HIV-1-RT antigen and CX3CL1-Ig promoted enhanced tumor rejection compared to DNA vaccine without CX3CL1-Ig [40].

Dorgham *et al.* identified a CCL5 analog (super-agonist) that has an increased capacity to engage CCR5 [38]. Aravindaram *et al.* delivered CCL5 cDNA into the vaccination site before human gpDNA (hgp100) vaccination [45], and continued to augment the antitumor effect by injecting viral vectors expressing mRNA for both CCL5 and hgp100. Their results showed a significant immune cell infiltration at the vaccination site and a strong anti-tumor response [45]. Inoculation of a new CCR5 mutant, 1P7-immunoglobulin (1P7-Ig), along with tumor DNA, resulted in an increased CD8^+^ T cell presence in the tumor beds and a better protection against tumor growth. These murine studies illustrate the potential benefit of using chemokines in DNA cancer vaccine preparations. 

### 3.3. Transforming Non-Immunogenic TAAs into Cancer Vaccines by Fusion with Chemokines

Another vaccine strategy is to exploit the fact that chemokines are internalized upon binding to their corresponding receptors on iDCs, thereby facilitating the delivery of accompanying antigens to APC for processing and presentation. Biragyn *et al.* generated genetically fused proteins consisting of inflammatory chemokines and TAA, where the chemokines serve as a carrier for the previously non-immunogenic TAA [53,54,77,78]. Once internalized along with the chemokine via the chemokine receptor, TAA presentation on DCs increases 100 to 10,000-fold [7,79,80] and results in the generation of protective antitumor immunity. This strategy was tested in murine lymphoma cell lines whose non-immunogenic variable region sequences (sFv) was genetically fused to chemokines CCL7, CXCL10 [54] and CCL20 [53]. Immunization with chemokine-sFv protein elicited a T-cell dependent antigen-specific protective antitumor immunity [54]. This response was dependent on the ability of chemokines to deliver the fused TAA to a chemokine receptor for internalization, whereas the recruitment of DCs alone to the site of antigen immunization by non-fused mixtures of chemokine and antigen was not sufficient to break the non-responsiveness to tumor antigen [54]. Therefore, this strategy can potentially be used in the same manner with any chemokine that binds to chemokine receptors present on iDCs (e.g., CCR1, CCR2, CCR5, and CCR6) [1], facilitating the efficient delivery of tumor antigens to MHC class I processing and cross-presentation pathway [81]. 

### 3.4. Whole Cell/Lysate Cancer Vaccines and Gene-Modified Tumor Vaccines: From Bench to Clinical Trials

Whole cell/cell lysate vaccines are prepared by irradiating or lysing autologous or allogeneic tumor cells [21]. They can be genetically modified further to express certain TAAs or other molecules [21]. This approach provides another way to include chemokines as adjuvants to increase vaccine immunogenicity. Zibert *et al*. created genetically modified leukemia/lymphoma vaccine to express CCL3 plus IL-2 or CCL3 plus GM-CSF [48]. Data showed that groups of mice receiving CCL3 plus IL-2 had 46% survival and the CCL3 plus GM-CSF group had 75% survival compared to 0% in the control group. Injection of CCL3 as a single agent showed 29% survival. These results were accompanied by enhanced effector cell infiltration in the tumor beds. Nomura *et al.* designed mouse fibrosarcoma and ovarian carcinoma cells to encode genes for CCL21, CCL19, or CXCL12 in the presence or absence of co-infection with GM-CSF and IL-2. Chemokine addition alone showed additive anti-tumor effect, while the combination of chemokine plus IL-2/GM-CSF boosted the response even further [52]. As CXCL12 is implicated in tumor pathogenesis [82], its future in immunotherapy is still being debated.

B16 melanoma cells engineered to stably express CCL21 chemokine elicited a robust effector T cell infiltration when used as a vaccine [47]. Li *et al.* used prostate cancer cells to develop a novel fusion gene using three common cancer gene epitopes: hPSM-hPAP-hPSA (“3P”) [49]. Fusing this gene construct with plasmid DNA coding for CCL21 (pCCL21-3P-Fc), the investigators introduced this construct into the B16FO melanoma cell line to create a genetically modified tumor vaccine. Injection of this vaccine into mice bearing melanoma tumors showed efficient tumor rejection and improved survival, with additional therapeutic benefits when the regimen was combined withanti-PD-L1 antibody administration. 

Inoue *et al.* evaluated the effect of adding either CCL5 or CCL17 to irradiated GM-CSF producing WEHI3B cells [50]. Addition of both chemokines in this study showed additional benefits in survival and tumor rejection, with significant CD4^+^ and CD8^+^ T cell infiltration in TME [50].

Based on the above studies, a Phase I clinical trial is evaluating the cell-based vaccine composed of irradiated tumor cells transduced with granulocyte-macrophage colony-stimulating factor (GM-CSF) and CD40-ligand (CD40L) genes, called the GM.CD40L vaccine, in the presence or absence of CCL21 in patients with lung cancer [58]. Another Phase I study of XCL1and IL-12gene-modified autologous neuroblastoma vaccine for relapsed/refractory neuroblastoma has been completed recently [59]. Additionally, a Phase I-II study is open for pediatric patients with advanced neuroblastoma using repeated immunization with gene-modified, IL-2/XCL1-secreting neuroblastoma tumor cell vaccines [58,60] (Table 3).

## 4. Exceptions to the Positive Effect of Chemokine Adjuvants in Tumor Vaccines

Even though chemokines are a promising adjunct to growing cancer vaccine protocols, some studies have also uncovered deleterious effects of adding chemokines to cancer vaccines. For example, the addition of CCL3 to GM-CSF producing glioma cells nullified the therapeutic effect of GM-CSF [51]. In another trial, the triple gp100 DNA + CCL21 DNA + IL2 vaccines failed to demonstrate a benefit over the dual gp100DNA + CCL21 DNA vaccine combination, while gp100DNA + IL-12 DNA vaccines showed some efficacy [46]. These observation highlights caution when choosing a combination of chemokine-cytokine vaccines. 

## 5. Future Perspective

**Scheme 1:**
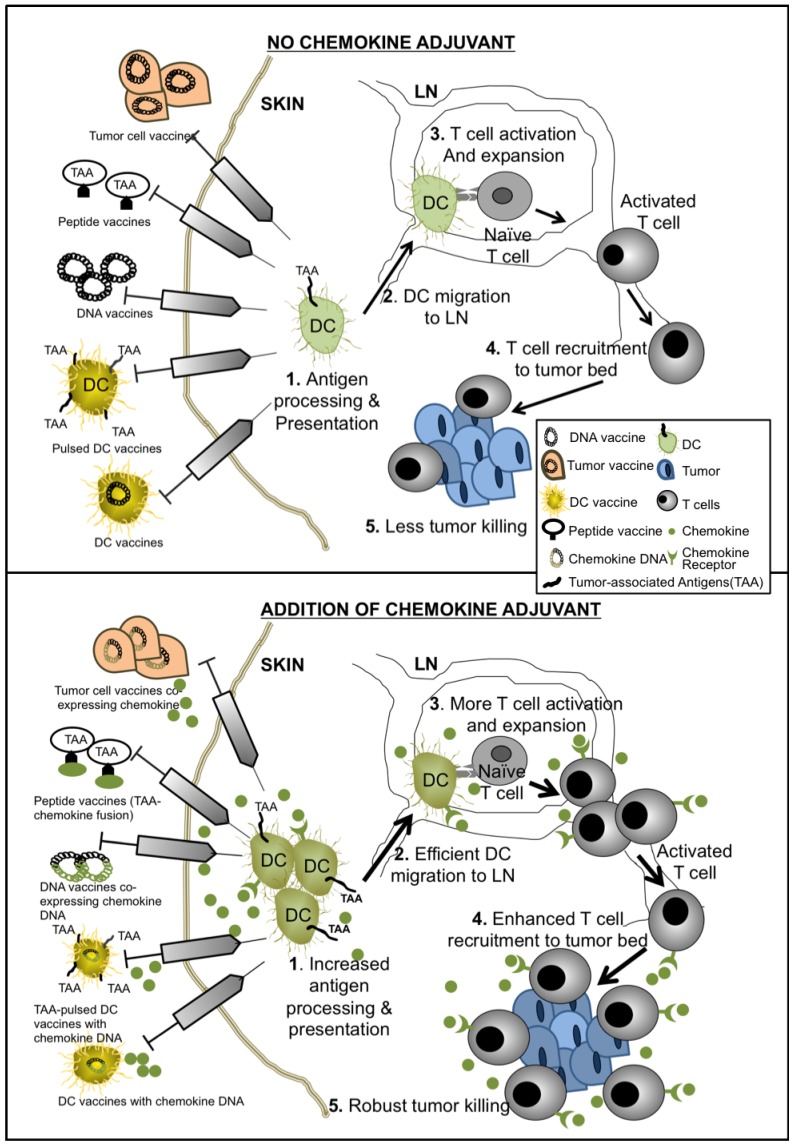
Mechanisms of chemokine-enhanced cancer vaccines.

The addition of chemokines into cancer vaccine strategies has the potential to provide great benefits in overcoming tumor tolerance by improving antigen presentation by APCs, enhancing effector cell priming, tumor eradication, and sustaining T cell memory responses (summarized in Scheme 1). Several studies have moved these concepts forward from pre-clinical studies into Phase I and Phase II trials in both adult and pediatric populations. Results of these highly anticipated trials would better inform investigators regarding next phases of new therapeutic development. Clearly, defining the precise disease stages (*i.e.*, bulky disease *versus* minimal residual disease) and timing during therapy when administering chemokine adjuvant therapy will be important next steps. Furthermore, characterizing which chemokine(s) to employ in various tumor types and the spectra of clinical scenarios in which to employ them will help to optimize the specific biological effects of these molecules for desired therapeutic outcomes. As cell-based immunotherapy (e.g., chimeric antigen receptor (CAR) lymphocyte therapies) [83,84,85] and immune checkpoint blockade (e.g., anti-PD1 and anti-PD-L1 antibodies) [86,87,88] approaches arrive at the forefront of novel cancer therapeutic development, the inclusion of relevant chemokines may further enhance therapeutic effectiveness by providing directed trafficking, accumulation and effector function delivery of therapeutic immune cells to the relevant body sites.

## 6. Conclusions

Evolving cancer vaccine strategies reflect our growing knowledge of tumor immunology, as classes of molecules (such as TLR agonists and chemokines) that are important in orchestrating effective host immune response find their way into various pre-clinical and clinical cancer vaccine and immunotherapy applications. An in-depth knowledge of the role of chemokines, cytokines and other biological agents will bring about their incorporation into vaccine preparations in the future to further boost therapeutic efficacy. In particular, current use of chemokines in cancer vaccines focuses on these molecules’ effect on migration and recruitment of relevant immune cells for effective antigen delivery and recognition. The potential additional effect of chemokines as direct functional co-stimulatory molecules on responding cells still remain largely unexplored. Future insights regarding the multi-faceted role of chemokines in immune response orchestration will further propel the field of cancer vaccine forward. In addition, it remains to be determined which vaccination strategies, timing and routes of administration involving chemokine adjuvants will be most efficacious in the clinical setting through well-designed clinical trials.
